# Highly compressed water structure observed in a perchlorate aqueous solution

**DOI:** 10.1038/s41467-017-01039-9

**Published:** 2017-10-13

**Authors:** Samuel Lenton, Natasha H. Rhys, James J. Towey, Alan K. Soper, Lorna Dougan

**Affiliations:** 1 School of Physics and Astronomy, University of Leeds, Leeds, LS2 9JT UK; 2 ISIS Facility, STFC Rutherford Appleton Laboratory, Harwell, Didcot, Oxford, OX11 0QX UK; 3 Present Address: Department of Biology, University of York, York, YO10, 5DD UK; 4 Department of Biochemistry, University of Oxford, South Parks Road, Oxford, OX1 3QU UK

## Abstract

The discovery by the Phoenix Lander of calcium and magnesium perchlorates in Martian soil samples has fueled much speculation that flows of perchlorate brines might be the cause of the observed channeling and weathering in the surface. Here, we study the structure of a mimetic of Martian water, magnesium perchlorate aqueous solution at its eutectic composition, using neutron diffraction in combination with hydrogen isotope labeling and empirical potential structure refinement. We find that the tetrahedral structure of water is heavily perturbed, the effect being equivalent to pressurizing pure water to pressures of order 2 GPa or more. The Mg^2+^ and ClO_4_^−^ ions appear charge-ordered, confining the water on length scales of order 9 Å, preventing ice formation at low temperature. This may explain the low evaporation rates and high deliquescence of these salt solutions, which are essential for stability within the low relative humidity environment of the Martian atmosphere.

## Abstract (web summary)

Significant amounts of different perchlorate salts have been discovered on the surface of Mars. Here, the authors show that magnesium perchlorate has a major impact on water structure in solution, providing insight into how an aqueous fluid might exist under the sub-freezing conditions present on Mars.

## Introduction

Water is widely regarded as a prerequisite for the development and survival of life because organisms require water for a range of biophysical and biochemical processes^1^. For instance proteins are surrounded by water in the form of a hydration shell that acts as a lubricant, facilitating the dynamic motion required for their function^2^. Furthermore, many biochemical reactions require water to be catalyzed by enzymes^3^. Hence, the search for extra-terrestrial life usually starts as a search for water. In this regard there is currently considerable interest in the growing evidence for the presence of flowing water on the surface of Mars^4–11^. Environmental conditions on the Martian surface are not suitable for pure water to remain as a liquid; however, the recent discovery of perchlorate in the regolith of the Martian northern plains has provoked some insight into how liquid water may exist below the Martian surface^4^. At high enough concentrations (~44 wt%) magnesium perchlorate salts can reduce the freezing temperature of water to as low as 206 K^6, 12–14^. An aqueous perchlorate solution could therefore provide the means for water to remain liquid, and therefore have flow at the sub-zero temperatures found on Mars^15^. In addition, water in the form of a briny liquid is likely to occur in other parts of the Solar System^16, 17^. Besides their potential extraterrestrial role, perchlorate solutions are also essential in many electrochemical studies since perchlorate holds the position of being the most chaotropic anion in the Hofmeister series^18^. Many of the unique properties of water are caused by the hydrogen bonding between water molecules and other constituents in the solution and it is well known that dissolved ions and cryopreservative molecules can significantly alter the structure of water in solution^19–24^.

One of the most direct ways to measure this structure in aqueous solutions is to use neutron total scattering experiments to investigate the hydrogen bond structure. Neutrons are used because they are strongly scattered by hydrogen atoms, whose contribution to the scattering pattern can be markedly altered by substituting deuterium for hydrogen. Hence, we study the structure of water in magnesium perchlorate solutions using total neutron scattering in combination with hydrogen isotope substitution. The data are interpreted with the computational modeling technique called “empirical potential structural refinement” (EPSR)^26–28^. Magnesium perchlorate has a eutectic point lower than many other perchlorate solutions, and is widely used as a mimetic in studies of Martian “briny” water^5^. Specifically our research focuses on 44 wt% solutions of magnesium perchlorate at 216 K, above the eutectic point of 206 K, and at room temperature 298 K.

Fundamentally our conclusions are that magnesium perchlorate has a major impact on water structure in solution. The relatively pronounced cation to anion association is mediated by water molecules and causes a major disruption to water structure. This disruption is the equivalent to a pressure in pure water needed for the formation of Ice VII. The first coordination shell in this form of ice is characterized by both hydrogen-bonded and non-hydrogen-bonded water molecules. To a marked extent water–water hydrogen bonds in the pure liquid are replaced by cation-water-anion bonds in solution. This, no doubt, is a major contributing factor to the very deep eutectic point of this solution. It may also explain the high deliquescence and low evaporation rates associated with this salt—factors that are important for the solutions to be stable under the low and widely varying humidity conditions found on the surface of Mars^10^.

## Results

### EPSR simulations

**Fig. 1:**
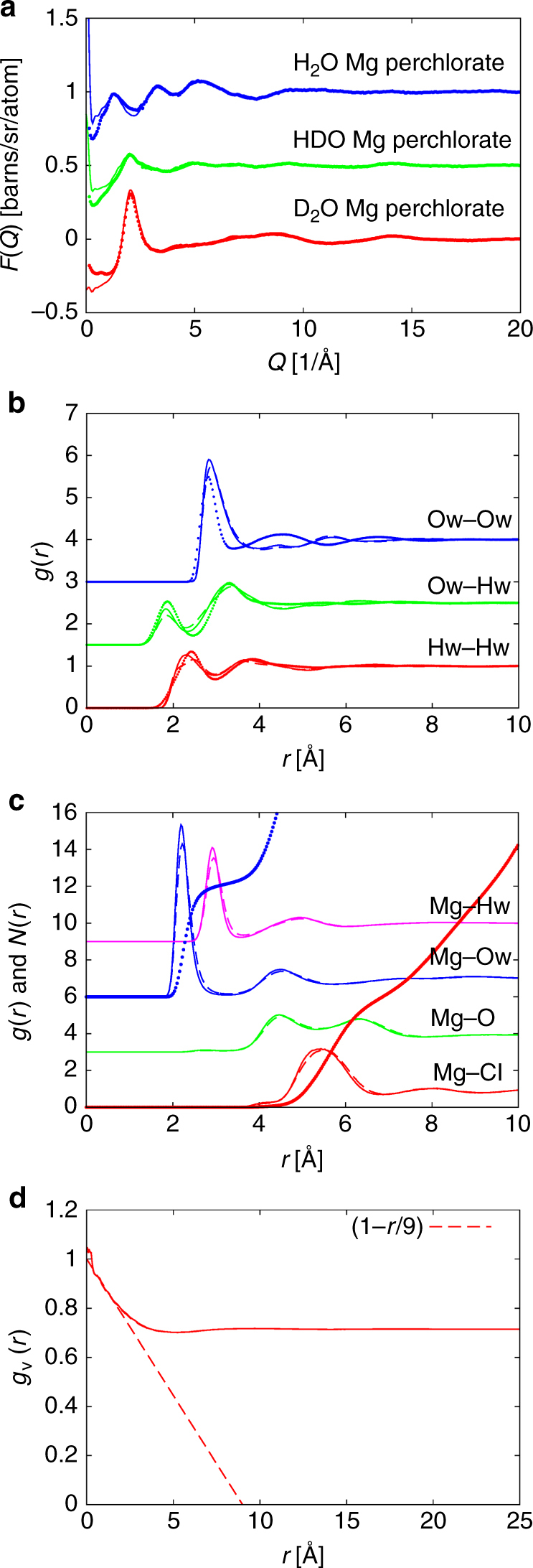
EPSR analysis of Mg(ClO_4_)_2_ 44 wt% aqueous solutions. **a** The measured total interference differential scattering cross-section, *F*(*Q*), at 216 K as a function of *Q* (dots) compared to the EPSR fits (lines) for different degrees of water deuteration, H_2_O (blue), D_2_O (red) and a 50:50 mole mixture of H_2_O and D_2_O (HDO) (green). **b** EPSR-simulated water–water site–site RDFs at 216 K (solid lines) and 298 K (dashed lines). Also shown are the same functions for pure water at 298 K (dots)^25^. **c** Simulated Mg—water (blue and pink) and Mg—perchlorate (red and green) RDFs at 216 K (solid lines) and 298 K (dashed lines). Also shown are the running coordination numbers of the same functions at 216 K. These do not change significantly at the higher temperature. **d** Water volume RDF at 216 K. This is indistinguishable from the same function at 298 K. The dashed line shows the approximate slope of this RDF at low *r*, indicating a confining length of the water of approximately 9 Å. In plots **a**–**c** the different functions are shifted vertically for clarity

For the EPSR computational modeling, a cubic simulation box was constructed at the same concentration, temperature and atomic number density as the experimentally measured samples, using relevant starting reference (seed) potentials and molecular structures^29–31^. Note that the measured neutron data for the three different isotopic mixtures are compared to the fits produced by the EPSR simulation in Fig. 1a.

### Water local structure

The EPSR simulation yields structural information on all the interactions in the solution, but here we focus on the key interactions concerning the structure of water and the ion–water interactions. The local structure of water in the magnesium perchlorate solutions is given by the radial distribution functions (RDFs) between the oxygen and hydrogen of water, Ow and Hw, respectively (Fig. 1b). By comparison with the same functions in pure water, it is seen that the solute in this case has a drastic effect on water structure, effectively eliminating the second peak in the Ow–Ow RDF at 4.5 Å. It is this peak that is widely regarded as indicating the underlying tetrahedral order in ambient water since it occurs at approximately $\sqrt {8/3}$ of the position of the first peak (2.8 Å)^32^. Equally the third peak in the bulk water Ow–Ow function at ~6.8 Å is drawn in to ~5.8 Å in the perchlorate solution.

### Ion-water structure

The magnesium to other-atom RDFs are shown in Fig. 1c. It should be mentioned that the neutron data are relatively insensitive to the how close the perchlorate ion can approach the oppositely charged magnesium ion. Two simulations were run either allowing magnesium-perchlorate contacts or not allowing such contacts. The latter case gave slightly better fits to the scattering data and is the one shown here. It can be seen from the running coordination numbers out to the first minimum in the corresponding RDF that the magnesium ion is surrounded by ~6 water molecules at a distance of ~2.2 Å, suggesting an octahedral arrangement of these water molecules on average. The hydrogen atoms of these molecules point away from the cation, as expected.

Although much further out at ~5.4 Å, the magnesium to perchlorate RDF (Mg–Cl) also shows a pronounced correlation with coordination number of ~5.8, suggesting there is a high degree of charge ordering in this solution. The Mg–O RDF shows a double peak, which straddles the Mg–Cl peak almost symmetrically.

### Water volume distribution function

Figure 1d shows the water volume distribution function (see Methods for more information). This is calculated by dividing the simulation box into a large number (~1 × 10^6^) of volume pixels, and assigning a value of “occupied” or “not-occupied” to each pixel. A pixel is regarded as occupied if it lies within 2.05 Å of a water oxygen atom, Ow. This value was chosen so that the fraction of occupied pixels corresponds to the volume fraction of the solution (~75%). Then a RDF for all the occupied pixels is calculated, called the volume RDF, *g*_v_(*r*). The long range part of this function corresponds to the volume fraction of the solution occupied by water—about 0.75 in the present case—while the initial decay of this function is indicative of the degree of local clustering of water molecules due to charge-ordering with an average confinement length of 9 Å^33–35^.

### Effect of ions on water structure

**Fig. 2:**
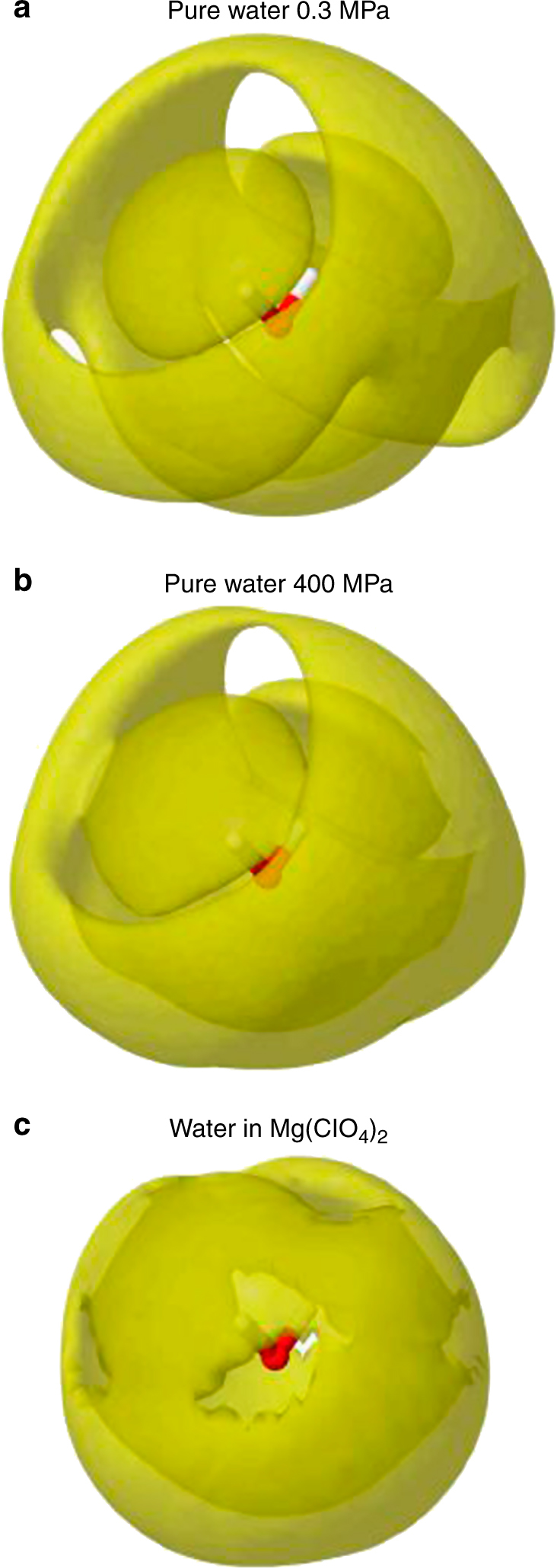
Spatial density functions of water around a central water molecule. Spatial density functions for **a** pure water at 268 K and 0.3 MPa, **b** pure water at 268 K and 400 MPa, and **c** water in Mg(ClO_4_)_2_ aqueous solutions at 216 K and 0.1 MPa. (The quoted pressures here refer to the pressures at which the scattering data were measured, not to the pressures of the EPSR refinements, which are constant volume simulations.) These surface contours enclose the highest density regions that contain 30% of the water molecules in the distance range 0–5 Å from a central molecule in the liquid. For pure water at ambient pressure, **a**, a first and second coordination shell are apparent, the second shell being roughly in anti-symmetry to the first shell. With application of pressure this shell collapses inwards but is still distinct (**b**). In Mg(ClO_4_)_2_ aqueous solutions the second shell has collapsed completely onto the first shell, **c**, in a manner reminiscent of Ice VII

The substantial effect of the magnesium perchlorate ions on water structure is demonstrated in Fig. 2, where the spatial density functions of pure water near ambient conditions and under pressure (400 MPa)^36^ are compared with the spatial density function of water in Mg perchlorate solutions found here. It can be seen that, as already anticipated, the water second coordination shell in pure water has completely collapsed onto the first shell, in a manner reminiscent of what happens when water is compressed to ~2 GPa to form Ice VII^37^. The fact that dissolved ions can modify water structure so dramatically has often been overlooked, but has been seen in a number of previous neutron studies^19, 38–40^.

**Fig. 3:**
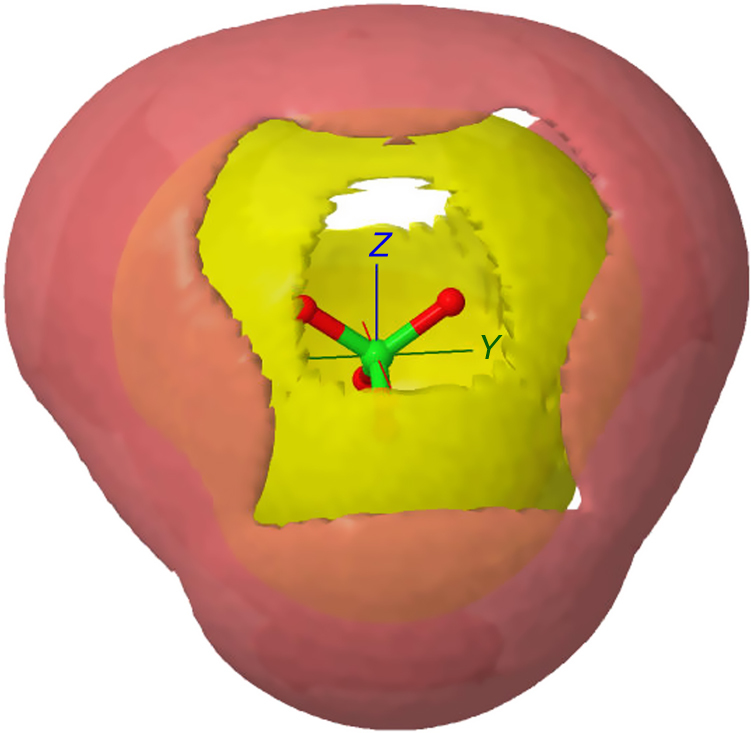
Distributions of water and magnesium around the perchlorate ion. Distribution of water (yellow) and magnesium (red) around the perchlorate ion in Mg(ClO_4_)_2_ aqueous solutions. For the water distribution the contour encloses 30% of the water molecules in the distance range 0–5 Å. For magnesium the contour encloses 30% of the magnesium atoms in the distance range 0–7 Å

To ascertain the nature of anion—water and anion—cation binding the spatial density function of Mg and water around the perchlorate anion is shown in Fig. 3. It is notable that both water and magnesium prefer to associate with the oxygen atoms of the perchlorate ion, the former through hydrogen bonding, while the latter is a purely Coulombic attraction. There is also some association along the edges of the perchlorate tetrahedron, but weaker bonding to the faces. The position of the magnesium second shell (red) further away from the cation than the water suggests that water acts as an intermediary between the anion and cation in this solution.

**Fig. 4:**
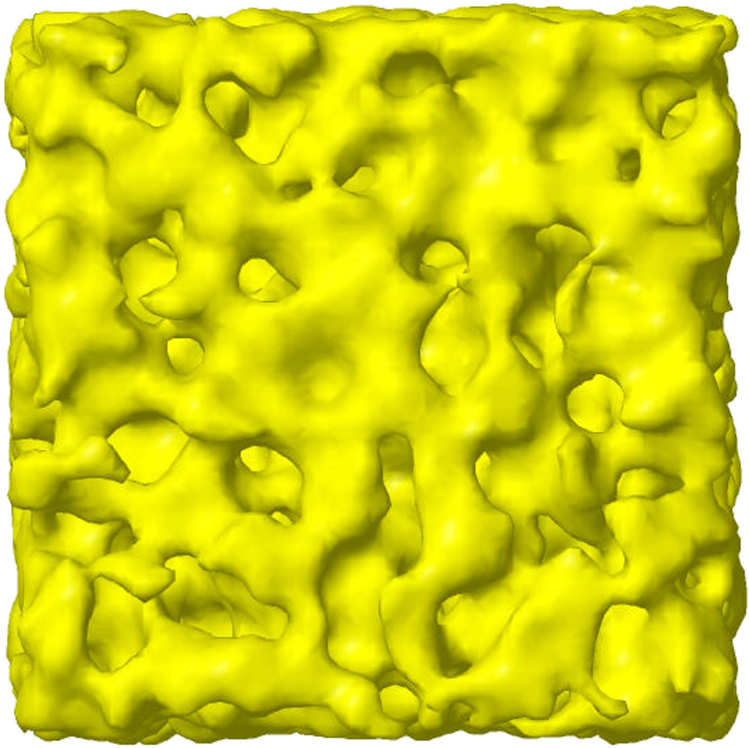
Distribution of water in Mg(ClO_4_)_2_ aqueous solutions. To generate the distribution of water in Mg(ClO_4_)_2_ aqueous solutions at 216 K, a Gaussian density function is placed at the center of each water oxygen atom in the EPSR simulation box with a RMS deviation (*σ*) of 1.5 Å (FWHM = 3.53 Å). A surface contour around the combined densities from all the water molecules is then drawn at half the maximum density. The length of one side of the cubic simulation box is 57.55 Å

Figure 1d indicates that the effect of the matrix of ions in solution gives rise to a confining length of order 9 Å in this system. This local confinement is visualized in Fig. 4, which shows the water density as a function of position through the EPSR simulation box. This distribution changes continually as the simulation proceeds, but the basic picture of a partially segregated water network remains unchanged throughout. Decreasing the temperature to 298 K does not change this picture significantly.

## Methods

### Neutron measurements

Measurements were performed on the SANDALS time-of-flight diffractometer at the ISIS neutron facility, covering a wide *Q*-range (0.2–30 Å^−1^). The neutron diffraction measurement yields the total interference differential scattering cross-section, *F*(*Q*), which is the sum of all partial structure factors, *S*_*αβ*_(*Q*), present in the sample each weighted by their composition, *c*, and scattering intensity, *b*, so that *F*(*Q*)* = ∑*_*αβ*_ *c*_*α*_ *c*_*β*_ *b*_*α*_ *b*_*β*_(*S*_*αβ*_(*Q*)−1), where *Q* is the magnitude of the change in the momentum vector by the scattered neutrons^12, 14, 16^. The *S*_*αβ*_(*Q*) are related by direct Fourier transform to the respective atom–atom RDFs *g*_*αβ*_(*r*). Integration of *g*_*αβ*_(*r*) yields the running coordination number of atoms *β* around *α* atoms, *N*_*αβ*_(*r*).

### Densities of Mg(ClO_4_)_2_ solutions

The density of the solutions was measured at room temperature using a densitometer. This value, 0.0958 atoms per Å^3^, was used both to the convert the raw scattering data to differential scattering cross-section per atom, and in the EPSR simulations. For the low-temperature solutions, a density was chosen so that the high *Q* differential scattering cross-section, which depends only on the level of single atom scattering, had the same value as at room temperature, and assuming the sample composition did not change at the lower temperature. This gave a value of 0.0973 atoms/Å^3^.

### EPSR simulations of Mg(ClO_4_)_2_ solutions

The EPSR simulations consisted of 318 Mg^2+^, 1 H^+^, 637 ClO_4_^−^ ions and 5015 water molecules placed in a cubic box of dimension 57.5532 Å at 216 K and 57.8520 Å at 298 K, giving atomic number densities of 0.0973 atoms/Å^3^ and 0.0958 atoms/Å^3^, respectively. (The small addition of perchloric acid was to chemically stabilize the solution by maintaining pH. This makes negligible contribution to both data and simulation.) This composition corresponds to the eutectic composition of 44 wt% Mg(ClO_4_)_2_. The water parameters are the same as the SPC/E water potential^41^ Coulomb interactions were truncated using the reaction field method described by ref. ^42^. The Lennard-Jones and Coulomb charge parameters for the seed potentials used for the simulations are given in Supplementary Table 1, and the fits obtained are shown in Supplementary Fig. 1.

For the perchlorate ion the values derive from those used in ref. ^29^ modified to use full electronic charges on the ions, while keeping the O–Ow nearest neighbor distance similar to the Ow–Ow nearest neighbor distance. For the perchlorate ion, a Cl–O bond distance of 1.42 Å with an O–Cl–O angle of 109.47° was observed, as in ref. ^30^. For magnesium, the initial guess for the potential parameters was adjusted to give the expected cation–water distance from ref. ^29^, namely, ~2.2 Å. In addition minimum allowed separations of 2.0 Å on Mg–Mg correlations, 3.3 Å on Mg–Cl correlations, 2.4 Å on Mg–O correlations, and 1.7 Å on Hw–Hw correlations were imposed on the EPSR simulations. The Mg–Cl and Mg–O minimum distances ensured contact ion pairs did not occur in these simulations. This gave marginally better fits than if contact ion pairs were allowed, but did not affect the primary observation of a dramatic effect on water structure in the presence of these ions.

### EPSR simulations of pure water at 268 K and 0.3 and 400 Mpa

In this case, since it would be impossible to measure pure water at 216 K, the scattering data were taken from an earlier publication^35^, which measured water structure at 268 K and at pressures up to 400 MPa. The EPSR simulations were run based on these existing data. The reference potential for these simulations was taken from a previous publication^43^ and the values are given in Supplementary Table 2. In each case the cubic simulation box contained 2000 water molecules, with box dimensions of 38.940 Å at 0.3 MPa and 37.456 Å at 400 MPa, corresponding to atomic number densities of 0.1016 atoms/Å^3^ and 0.1142 atoms/Å^3^, respectively.

### Volume RDF

This function is used as device to understand the degree of confinement of a fluid in an absorbing matrix. Reference ^34^ gives a recent example of an application of this method. The idea derives from earlier methods appropriate to the analysis of small-angle scattering data. In essence the computer simulation box is divided into a larger number of volume pixels. Each pixel is listed as either occupied or not-occupied depending on how far it is from any water molecule. A pair correlation function for all the occupied pixels is then generated as a function of their separation, *r*, in the usual way using the minimum image convention, and this is normalized to go to unity at *r* = 0. The large *r* value of this correlation then corresponds to the volume fraction of occupied pixels. The choice of distance to specify whether a pixel is occupied is in principle arbitrary, except that we know independently from the atomic number density and the solution concentration what fraction of the volume of solution is occupied by water. At the present concentration of Mg(ClO_4_)_2_ this water volume fraction is ~0.75, so to achieve this value in the final volume correlation function the maximum distance from a water molecule for a pixel to be occupied was set to 2.05 Å. Following Glatter and Kratky^32, 33^, the initial decay of this function is a measure of the confining dimension of the water.

### Data availability

Data associated with this work are available from the Research Data Leeds repository under a CC-BY license at: https://doi.org/10.5518/210.

## Electronic supplementary material

Supplementary Information
