# Supplementary material for: Highly compressed water structure observed in a perchlorate aqueous solution
Source: Nat Commun. 2017 Oct 13;8:919. doi: 10.1038/s41467-017-01039-9 (PMC5715023; doi:10.1038/s41467-017-01039-9)
Supplement: Supplementary file 1 — Supplementary Information [file 41467_2017_1039_MOESM1_ESM.pdf]

Supplementary Figure 1. EPSR analysis of $Mg(ClO_4)_2$ 44 wt % aqueous solutions. The measured total interference differential scattering cross section, F(Q), at 298 K as a function of Q (dots) compared to the EPSR fits (lines) for different degrees of water deuteration, H_2O (blue), D_2O (red) and a 50:50 mole mixture of H_2O and D_2O (HDO, green).

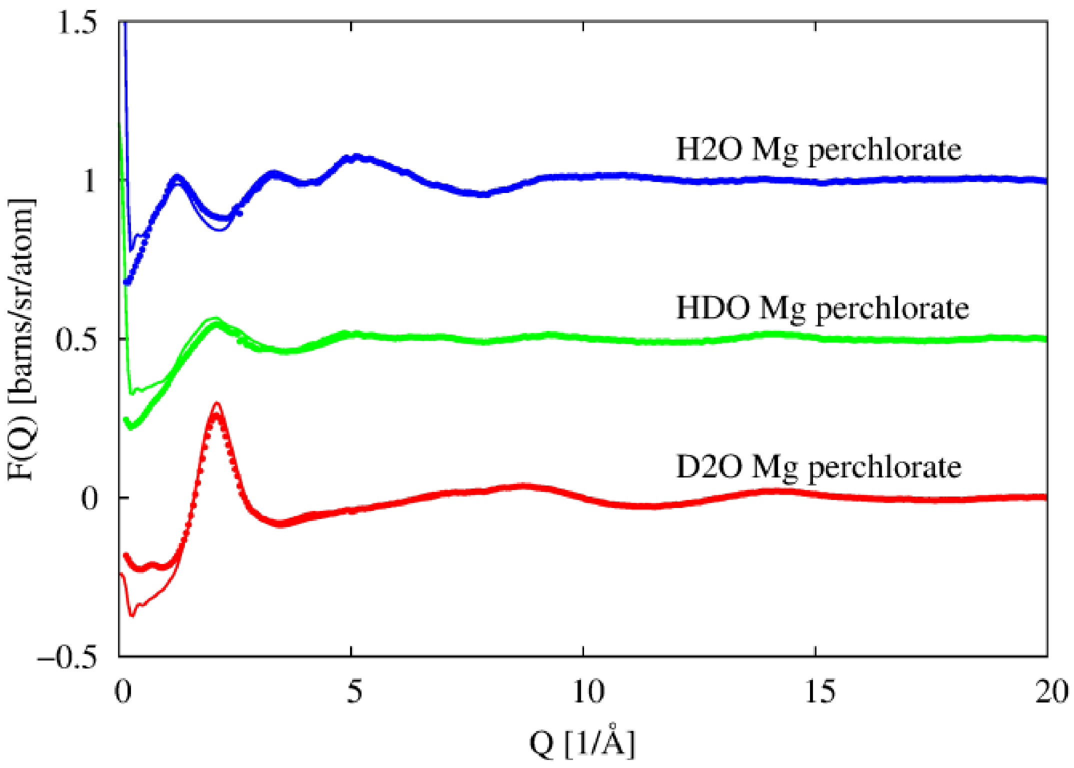

Supplementary Table 1. Lennard-Jones and Coulomb charge (*q*) parameters used in EPSR simulation. Ow and Hw refer to water atoms.

Atom type	ε [kJ/mole]	σ [Å]	q [e]
Mg	0.4593	0.90	+2.0000
Cl	0.5660	4.19	+2.3904
O	0.6500	3.166	-0.8476
H	0.7500	0.000	+1.0000
Ow	0.6500	3.166	-0.8476
Hw	0.0000	0.000	+0.4238

Supplementary Table 2. Lennard-Jones and Coulomb charge (*q*) parameters used in EPSR simulations of pure water. Ow and Hw refer to water atoms.

Atom type	ε [kJ/mole]	σ [Å]	q [e]
Ow	0.300	3.2	-1.0
Hw	0.0000	0.000	+0.5
